# Impact of In-Person and Mobile Exercise Coaching on Psychosocial Factors Affecting Exercise Adherence in Inactive Women With Obesity: 20-Week Randomized Controlled Trial

**DOI:** 10.2196/68462

**Published:** 2025-02-25

**Authors:** Christina Gjestvang, John Magne Kalhovde, Elene Mauseth Tangen, Hege Clemm, Lene Annette Hagen Haakstad

**Affiliations:** 1 Department of Sports Medicine Norwegian School of Sport Sciences Oslo Norway; 2 Department of Health and Exercise School of Health Sciences Kristiania University of Applied Science Oslo Norway

**Keywords:** barriers, exercise behavior, exercise coaching, health-related quality of life, motivation, obesity, psychosocial factors, self-efficacy, social support

## Abstract

**Background:**

Regular exercise may counteract obesity-related health risks, but adherence is low among individuals with obesity. Personal trainers may positively influence exercise behavior by providing motivational support. Individuals who receive regular exercise coaching are more likely to adhere to their exercise routine, compared with those who exercise individually. However, investing in personalized exercise guidance, such as a personal trainer, can be expensive for the individual. Thus, integrating web-based coaching could be a more economically sustainable option, offering both flexibility and reduced costs compared with in-person coaching only. Yet, research is needed to assess the effect of hybrid models in improving psychosocial factors among women with obesity.

**Objective:**

This 20-week, pragmatic randomized controlled trial aimed to investigate the effect of weekly in-person coaching compared with 2 combinations of in-person and web-based coaching on 5 psychosocial factors in women with obesity (BMI ≥30 kg/m^2^).

**Methods:**

Participants were invited through Facebook and Instagram advertisements posted by various fitness clubs across rural and urban locations in Norway (7 different counties and 12 different municipalities). Women with low activity (n=188; <150 minutes of moderate-intensity physical activity/week; 42.7, SD 10.5 years; mean BMI of 35.1, SD 6.9 kg/m^2^) were allocated into 3 groups of in-person coaching—weekly (n=47), twice weekly (n=47), and once monthly (n=47)—and as controls (n=47). Those with twice weekly and once monthly in-person coaching received web-based coaching of 15 minutes during weeks without in-person coaching. Data included background variables, motivation (Behavioral Regulation in Exercise Questionnaire-2), barriers, self-efficacy (The Self-Efficacy Survey), social support (Social Support Questionnaire), and health-related quality of life (36-Item Short Form Health Survey [SF-36]).

**Results:**

A total of 120 (64%) out of 188 participants completed baseline and postintervention assessments. A minor difference was observed in one item of the SF-36, where all intervention groups reported a greater “change in health last year” than the control group (mean difference: 14.2-17.1, 95% CI 2.04-29.5; *g*=0.79-1.14; *P*≤.01). No other effects were found on the selected psychosocial factors. It should be noted that controls reported higher intrinsic motivational regulation at baseline than intervention groups (mean difference: 0.43-0.93; *P*≤.05). All intervention arms exercised more frequently than controls (mean difference: 1.1-1.5; *P*≤.001), with no differences in weekly exercise frequency between the 3 intervention arms (*P*=.30).

**Conclusions:**

We found no effects on motivation, barriers, self-efficacy, perceived social support, or other health domains compared with controls. All intervention groups reported a slight improvement in self-perceived health in 1 of the 8 subscales of the SF-36. Combined in-person and web-based coaching may give a minor improvement in self-perceived health in women with obesity. However, the lack of impact on motivation, barriers, and self-efficacy warrants further research.

**Trial Registration:**

ClinicalTrials.gov NCT05792657; https://clinicaltrials.gov/study/NCT05792657

## Introduction

Obesity (BMI ≥30 kg/m^2^) is a major global health challenge, significantly economically burdening the health care and welfare systems [1,2]. Worldwide, the obesity rate has nearly tripled in the past 35 years, and it is estimated that 1 billion adults will have obesity in 2030 [3-5]. It affects women more than men, with 1 in 5 women and 1 in 7 men living with obesity [5,6]. Obesity increases the risk of noncommunicable diseases, such as cardiovascular disease, metabolic disease, and type 2 diabetes [7-11]. It is also negatively associated with mental health conditions, including anxiety, personality disorders, binge eating, and schizophrenia [12].

Structured exercise programs have become a key element in obesity management worldwide, offering benefits for both physiological and psychological well-being [13-15]. Regular exercise can mitigate obesity-related health risks and is widely recommended, along with diet advice and behavioral strategies, as part of a comprehensive treatment strategy [9,11,16-18]. The feasibility of these programs is frequently challenged by barriers such as time constraints, logistical issues, and accessibility, particularly for women with obesity [13,14]. In addition, the complexity of implementing lifestyle changes often results in low adherence rates, which limits the associated health benefits [19,20]. This challenge is further compounded by the fact that individuals with obesity tend to be more sedentary and less active than those of normal weight, and women are less likely than men (32% versus 23%) to meet physical activity recommendations (150 to 300 minutes of moderate-intensity or 75 to 150 minutes of vigorous-intensity physical activity per week) [21-24].

Low levels of motivation, limited strategies to overcome barriers, lack of self-efficacy, inadequate social support, and poor physical or mental health negatively impact an individual’s ability to maintain regular exercise [19,25-28]. Exercise coaching may positively influence these factors by fostering autonomy (eg, offering choices and alternatives), building competence (eg, providing positive feedback and promoting skill development), and improving relatedness (eg, offering emotional support) [28-31]. As a result, these elements collectively have the potential to improve adherence to exercise [28,32-34]. Randomized controlled trials (RCTs) have shown that personal trainers help clients adhere more consistently to exercise by equipping them with strategies to overcome barriers, fostering a sense of community, and enhancing self-efficacy through knowledge and confidence building [31,32,35-37].

Further, perceived social support has been shown to encourage regular participation in exercise, particularly among women rather than men [25,38,39]. In addition, obesity is often associated with lower self-perceived health, and individuals who perceive their health as poor may face physical limitations that hinder participation in regular exercise [27,40]. A personal trainer can develop customized exercise programs that address these physical limitations, helping clients overcome barriers and ultimately improve their perceived health-related quality of life [41,42].

Although individuals with obesity might acknowledge the benefits of personal trainers in providing exercise motivation and support, their use is often limited by barriers such as accessibility, time constraints, socioeconomic resources, and cost concerns [43-45]. The high cost of hiring a personal trainer also often limits the frequency of follow-up sessions. Mobile exercise apps have emerged as a popular and accessible alternative [14]. When used in conjunction with or as an extension of qualified exercise professionals, mobile apps may enhance exercise adherence [14]. Hence, integrating web-based coaching is an attractive alternative due to its cost-effectiveness and accessibility. Web-based coaching offers flexibility and lower costs compared with in-person coaching [46,47], while in-person coaching provides unique advantages, such as fostering deeper personal relationships that may be vital for sustained exercise adherence. Consequently, hybrid models combining in-person and web-based coaching have gained popularity, as they provide the flexibility and convenience of web-based coaching with the direct interaction and personalized feedback of in-person coaching. These models show potential for promoting behavior change by meeting a wider range of client needs. However, the effectiveness of such combined approaches, particularly for women with obesity, requires further investigation [46,47]. Hence, further research is needed to evaluate the potential of hybrid models in addressing key psychosocial factors to exercise adherence, particularly for women with obesity. While these models are promising in combining accessibility with personalized support, their ability to positively influence motivation, barriers, self-efficacy, social support, and health-related quality of life remains unclear. In addition, to provide a “real-life” approach, weekly in-person coaching is needed as a comparison group, as it represents the most common mode of in-person coaching among clients [48]. Understanding how such hybrid models can optimize psychosocial outcomes in this population is crucial for developing effective and sustainable interventions.

In women with obesity, our 20-week, 4-armed, pragmatic RCT aimed to investigate the effects of weekly in-person coaching compared with 2 different combinations of in-person and web-based coaching on motivation, barriers, self-efficacy, social support, and health-related quality of life.

## Methods

### Overview

This 20-week, 4-armed, pragmatic RCT’s primary outcomes were exercise adherence, aerobic capacity, and muscular strength. Hence, this study presents a secondary analysis of the intervention’s effect on perceived motivation, barriers to exercise, self-efficacy, social support, and health-related quality of life.

Participants were invited through Facebook and Instagram advertisements posted by various fitness clubs across rural and urban locations in Norway (7 different counties and 12 different municipalities). The recruitment was ongoing in February 2023, with eligible participants enrolled continuously. Unfortunately, data on the total number of interested participants were not collected. The inclusion criteria were BMI ≥30 kg/m^2^, aged 18 to 65 years, no membership in a fitness club 6 months before recruitment, being classified as low activity (<150 minutes of moderate-intensity or 75 minutes of vigorous-intensity physical activity per week), Norwegian speaking, and having a smartphone. Exclusion criteria were chronic disease or injuries (eg, severe hypertension 180/110 mm Hg, heart disease, lung disease, or functional impairment) that hindered participation in exercise and planned leave during the intervention period. To ensure that none of the participants had any contraindications for exercise, all participants underwent a general health screening by their general practitioner.

A total of 188 participants were included, and informed consent was signed to participate. A blinded statistician performed random allocation (1:1:1:1) following a simple computer-based randomization program (R for Windows, version 4.2.2; R Foundation for Statistical Computing), and the participants were allocated to (1) once a week (in-person exercise coaching 100% [IP100], n=47), (2) twice a month (in-person exercise coaching 50% [IP50], n=47), or (3) once a month (in-person exercise coaching 25% [IP25], n=47) in-person coaching, or as controls (n=47). The intervention groups were provided the same weekly frequency of follow-up, but in IP50 and IP25, a total of 50% (2/4) and 75% (3/4) of the follow-up sessions were replaced with 15-minute digital sessions, respectively. An overview of intervention arms is shown in Textbox 1.

**Overview of intervention arms in this 20-week, pragmatic randomized controlled trial.**

**In-person exercise coaching 100% (IP100)**

- One weekly in-person exercise coaching.
- A total of 20 hours of in-person coaching.
- Access to the ABEL-app (Abel Technologies AS) including individual exercise programs, progress charts, nutritional advice, and motivational notices.
- 20 weeks fitness club membership.

**In-person exercise coaching 50% (IP50)**

- Two monthly in-person exercise coaching and 15 minutes of web-based coaching during weeks without in-person coaching.
- Total of 10 hours of in-person coaching during the intervention.
- Access to the ABEL-app including individual exercise programs, progress charts, nutritional advice, and motivational notices.
- 20 weeks fitness club membership.

**In-person exercise coaching 25% (IP25)**

- One monthly in-person exercise coaching and 15 minutes of web-based coaching during weeks without in-person coaching.
- Total of 5 hours of in-person coaching during the intervention.
- Access to the ABEL-app including individual exercise programs, progress charts, nutritional advice, and motivational notices.
- 20 weeks fitness club membership.

**Control group**

- Was asked to continue with normal life.
- Received the “Norwegian Directorate of Health’s” recommendations for physical activity and nutrition.
- Access to the ABEL-app to register physical activity and exercise, but were not provided with individual exercise programs, nutritional advice, and motivational notices.

A total of 23 exercise professionals, all employed as personal trainers, were recruited to provide follow-up for study participants. Trainers needed at least 3 years of part-time experience and to have led at least 80 exercise sessions/month recently. Of the 23 trainers, 10 had acquired certification as personal trainers, 8 held relevant bachelor’s or master’s degrees in fields such as exercise science or physiotherapy, and 5 had bachelor’s or master’s degrees in health and social care education. The trainers were instructed to follow up with each participant and create customized weekly exercise programs based on their needs. They also offered lifestyle advice, such as coaching in maintaining a healthy diet. Our study design did not allow for further masking of study participants or the personal trainers.

### Measures

#### Overview

A standardized electronic survey was used to obtain information about exercise motivation, barriers to exercise, self-efficacy, social support, and health-related quality of life, as well as background variables (body weight and height, age, education, household income, and occupation). The survey took approximately 20 minutes to complete, with all questions being close ended. Of the 188 participants, 120 (64%) participants completed the questionnaire at both baseline and after 20 weeks (postintervention) and were included in the current analysis.

#### Exercise Motivation

The measurement of exercise motivation was based on a Norwegian version of the validated Behavioral Regulation in Exercise Questionnaire-2 (BREQ-2) [49], assessing the stages of the self-determination continuum of motivation. The BREQ-2 comprises 5 subscales, with a total of 19 statements. For each statement, the participants rated the significance of each statement as a personal motive to engage, or not engage in exercise, on a 5-point scale (from “0 not true for me” to “4 very true for me”). A sum score (from 0 to 4) for each subscale was calculated by adding scores from each statement, divided by the number of statements. The BREQ-2 has good internal consistency for all 5 subscales (Cronbach α >0.7) [49,50]. In our study, Cronbach α values for the 5 subscales were as follows: 0.81 and 0.87 (intrinsic regulation), 0.69 and 0.80 (identified regulation), 0.73 and 0.80 (introjected regulation), 0.83 and 0.81 (external regulation), and 0.82 and 0.79 (amotivation) for before and after the intervention, respectively.

#### Barriers to Exercise

Barriers to exercise were assessed based on factors identified in a large-scale study among the Norwegian adult population [51] and a previous study among fitness club members [52]. The questionnaire section contained 18 barriers categorized into 4 subscales. The participants rated how limiting they perceived each barrier to be on a 3-point scale, ranging from “1 not important to me” to “3 very important to me.” By adding the score from each barrier divided by the number of statements, a sum score (from 1 to 3) for each subscale was calculated. The barrier subscales in the previous large-scale Norwegian study [51] have been shown to have Cronbach α values above 0.70 for the practical, health-related, and affective-cognitive subscales, but lower for the priority subscale. In this study, Cronbach α values for the 4 subscales were as follows: 0.53 and 0.50 (priority), 0.46 and 0.51 (practical), 0.22 and 0.39 (health-related), and 0.61 and 0.63 (affective-cognitive) for before and after the intervention, respectively. As expected, Cronbach α was lowest for subscales with the fewest items, and these subscales should be interpreted with care. Internal consistency of the whole barrier questionnaire was higher, as determined by Cronbach α as follows: 0.66 at baseline and 0.72 at posttest.

#### Self-Efficacy

An abbreviated and validated version of the Self-Efficacy Survey, comprising 2 subscales and a total of 12 statements, was used to measure self-efficacy for exercise [53]. The participants rated each statement on a 5-point scale (from “1 I know I cannot” to “5 I know I can”) in how confident they were to increase or continue with regular exercise under a wide range of conditions. For each subscale, a sum score (from 1 to 5) was calculated by adding the scores of each statement, divided by the number of statements. The Self-Efficacy Survey has been shown to have good test-retest reliability and internal consistency [53]. In our study, Cronbach α values for the 2 subscales were as follows: 0.89 and 0.90 (“sticking to it”), and 0.63 and 0.55 (“making time for exercise”) for before and after the intervention, respectively. Cronbach α was lowest for the subscale with 4 items and this subscale should be interpreted with care. The internal consistency of the entire Self-Efficacy Survey was high, as determined by the Cronbach α are as follows: 0.80 at baseline and 0.90 at posttest.

#### Social Support

Social support for exercise was based on a validated survey developed by Sallis et al [54], consisting of 13 statements. The participants rated each statement on a 5-point scale, ranging from “1 none” to “5 very often,” in how often their family or friends had been supportive of them exercising. A total social support score was calculated (from 13 to 65) by adding the scores from each of the statements, where higher scores demonstrated greater social support for exercise. The 2 sections (family and friends) were merged. The original survey has been shown to have acceptable test-retest reliability and internal consistency [54]. In our study, Cronbach α values for the 13 statements concerning social support were 0.83 and 0.87 at baseline and posttest.

#### Health-Related Quality of Life

The 36-Item Short Form Health Survey (SF-36) was used to measure subjective evaluations of health-related quality of life. The questionnaire encompasses 8 health-related subscales with 2 to 10 questions or statements and a single item that provides an indication of perceived change in health last year. Each set of response options is designed to quantify the intensity of the participant’s feelings or limitations of health and well-being [55]. As recommended, we precoded all items on a 0 to 100 range with a high score presenting a more favorable health state. A sum score for each subscale was then calculated by adding scores from each statement, divided by the number of statements. A Norwegian version of SF-36 has been shown to have good internal consistency for all subscales (Cronbach α >0.80) [56]. In our study, Cronbach α values for the 8 subscales pre/post was as follows: 0.84 and 0.85 (“physical functioning”), 0.84 and 0.90 (“role limitations due to physical health”), 0.84 and 0.86 (“role limitations due to emotional problems”), 0.85 and 0.19 (energy/fatigue), 0.71 and 0.31 (“emotional well-being”), 0.76 and 0.71 (“social functioning”), 0.88 and 0.82 (pain), and 0.71 and 0.79 (“general health”) for before and after the intervention, respectively. The internal consistency of the entire questionnaire section was high, as determined by Cronbach α, were as follows: 0.91 at baseline and 0.93 at posttest.

#### Participation in Exercise and Physical Activity

Data on exercise and physical activity participation were collected using the ABEL app (Abel Technologies AS). Information, including type of activity, intensity, duration, and frequency, was recorded by participants during individual sessions and by personal trainers during follow-up sessions.

### Statistical Analysis

The analyses were performed with SPSS statistics (version 28; IBM Corporation), following a predefined analysis plan and before unmasking the intervention arms. The normal distribution of the data was assessed with the Shapiro-Wilk test. We used appropriate statistical tests to compare baseline characteristics between the current sample and those who dropped out, as well as to identify differences between groups. These included a chi-square test, an independent 2-tailed *t* test, or a 1-way ANOVA, as dictated by the data. Postintervention, group comparisons were done using a 1-way ANOVA with Bonferroni post hoc comparisons. We also calculated Hedges *g* effect size, with values around 0.2, 0.5, and 0.8 interpreted as weak, medium, and strong effect sizes, respectively. Data are presented as mean (SD), numbers (%), mean changes, mean differences, 95% CI, effect sizes, and *P* value. The level of statistical significance was set at *P*≤.05 and adjusted as appropriate for the 1-way ANOVA and Bonferroni post hoc comparison (*P*≤.01).

In this study, targeting a specific population of women with obesity who have low activity presented challenges in accurately estimating attrition rates due to limited comparable data. Given the lack of reliable data on expected dropout rates within this demographic, the research group assumed recruitment of 200 participants was appropriate.

### Ethical Considerations

The study was conducted following the ethical standards of the Helsinki Declaration (as revised in 2000) and relevant national and institutional guidelines. All participants provided written informed consent after receiving detailed study information, including their rights to withdraw at any time without consequence. The study was reviewed by the Regional Committee for Medical and Health Research Ethics (REK 2022/552476), which determined that, under the Act on Medical and Health Research (the Health Research Act 2008), full review by the Regional Committee for Medical and Health Research Ethics was not required. Ethical approval was granted by the Norwegian School of Sports Sciences Ethical Committee (Applications number 262). The study was also registered with the Norwegian Social Science Data Service (Sikt 104437) and ClinicalTrials.gov (NCT05792657).

To protect participant privacy, no identifying information was collected or published. Participants in the intervention arms received regular follow-ups by a personal trainer, while all study groups, including the control group, had access to a fitness club and an exercise app (ABEL Technologies). No financial compensation was provided to participants.

## Results

**Figure 1:**
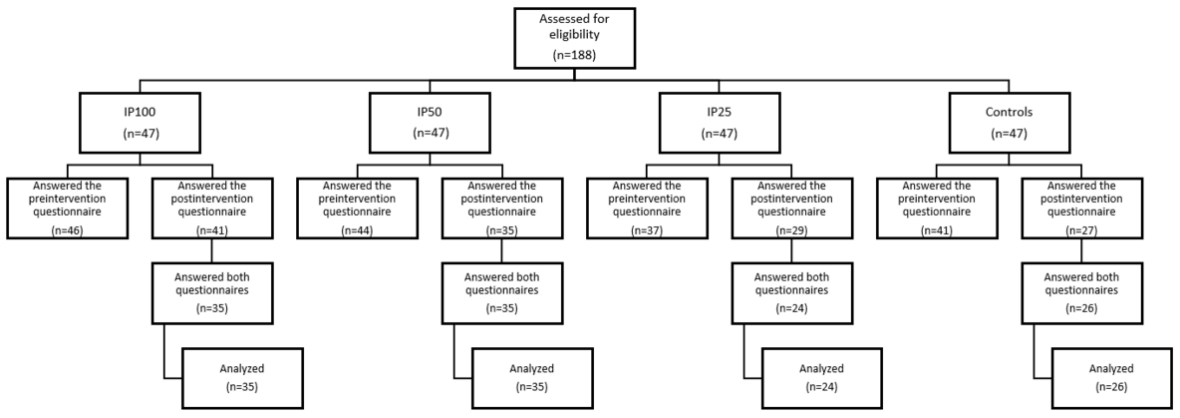
CONSORT (Consolidated Standards of Reporting Trials) flowchart of the participants. IP100: in-person exercise coaching 100%; IP25: in-person exercise coaching 25%; IP50: in-person exercise coaching 50%.

A total of 120 (64%) out of 188 participants completed baseline and postintervention assessments (IP100: n=35; IP50: n=35; IP25: n=24; and controls: n=26). The flow of the participants is shown in Figure 1. Mean age of the participants collapsed was 42.7 (SD 10.5) years and the mean BMI was 35.1 (SD 6.9) kg/m^2^. There were no differences between the 4 groups in age, body weight, education, or household income at baseline (all *P*>.05; Table 1). In terms of BMI, participants in the control group had a higher mean BMI (35.8, SD 5.8 kg/m^2^) than those allocated to IP100 (32.8, SD 2.6 kg/m^2^; *P*=.02). There were no significant differences between the current sample and study dropouts in background characteristics at baseline (data not shown).

Weekly exercise frequency over the 20-week intervention was as follows: 2.0 (SD 1.3) days/week (IP100), 2.4 (SD 1.5) days/week (IP50), 2.2 (SD 1.6) days/week (IP25), and 0.9 (SD 1.6) days/week (controls). We found no differences in weekly exercise frequency between the 3 intervention arms (*P*=.30); however, all intervention arms exercised more frequently than controls (*P*≤.001).

**Table 1:** Background characteristics, exercise motivation, barriers to exercise, self-efficacy, social support, and health-related quality of life at baseline in the 3 intervention arms and the control group.

		IP100^a^ (n=35)	IP50^b^ (n=35)	IP25^c^ (n=24)	Controls (n=26)	*P* values
Body weight (kg), mean (SD)		94.0 (9.8)	98.4 (12.6)	99.7 (10.6)	97.3 (14.5)	.28
Body height (cm), mean (SD)		169.1 (6.2)	167.5 (5.6)	168.1 (4.0)	164.9 (5.7)	.03
Age (years), mean (SD)		45.3 (10.5)	42.6 (10.2)	43.3 (11.4)	41.1 (9.4)	.46
BMI (kg/m^2^), mean (SD)		32.8 (2.6)	35.0 (3.9)	35.3 (3.5)	35.8 (5.8)	.02
Higher education (≥4 years), n (%)		7 (20.0)	9 (25.7)	8 (33.3)	10 (38.5)	.71
High household income (>US $75,000 per year), n (%)		22 (62.9)	19 (54.3)	11 (45.8)	12 (46.2)	.24
Employed outside home, n (%)		33 (94.3)	31 (88.6)	16 (66.7)	21 (80.8)	.01
**Exercise motivation, mean (SD)**						
	Intrinsic regulation	1.7 (0.9)	2.1 (0.6)	2.1 (0.8)	2.6 (0.7)	≤.001
	Identified regulation	2.1 (0.9)	2.2 (0.8)	2.1 (0.8)	2.4 (0.7)	.45
	Introjected regulation	1.8 (1.1)	2.0 (1.0)	1.7 (1.1)	2.0 (1.1)	.60
	External regulation	0.6 (0.8)	0.7 (0.8)	0.6 (0.9)	0.7 (1.0)	.91
	Amotivation	0.4 (0.6)	0.3 (0.7)	0.4 (0.7)	0.2 (0.5)	.70
**Barriers to exercise, mean (SD)**						
	Priority	1.9 (0.6)	1.8 (0.6)	1.8 (0.7)	1.7 (0.4)	.35
	Practical	1.4 (0.4)	1.5 (0.4)	1.4 (0.4)	1.5 (0.4)	.45
	Health-related	1.5 (0.4)	1.4 (0.4)	1.5 (0.4)	1.5 (0.4)	.69
	Affective-cognitive	1.5 (0.3)	1.4 (0.3)	1.4 (0.3)	1.5 (0.3)	.39
**Self-efficacy, mean (SD)**						
	Sticking to exercise	3.4 (0.9)	3.6 (0.9)	3.3 (0.9)	3.8 (1.1)	.06
	Making time for exercise	3.3 (0.8)	3.5 (0.9)	3.2 (1.0)	3.4 (1.0)	.39
Social support, mean (SD)		26.7 (7.2)	26.3 (5.3)	26.7 (1.6)	25.8 (1.2)	.87
**Health-related quality of life, mean (SD)**						
	Physical functioning	75.1 (13.4)	70.5 (16.0)	69.1 (18.2)	68.5 (22.9)	.29
	Role limitations due to physical health	69.3 (35.4)	67.9 (36.2)	58.3 (47.0)	60.6 (41.3)	.86
	Role limitations due to emotional problems	57.1 (46.1)	59.0 (40.5)	61.1 (43.6)	53.8 (42.2)	.86
	Energy/fatigue	40.6 (10.0)	37.5 (10.2)	39.2 (8.2)	33.8 (12.9)	.16
	Emotional well-being	59.9 (15.2)	53.6 (15.3)	61.6 (17.0)	51.8 (18.1)	.10
	Social functioning	66.1 (30.7)	61.4 (31.3)	61.0 (30.5)	59.6 (33.4)	.66
	Pain	70.3 (20.8)	67.6 (26.1)	62.4 (25.3)	59.4 (25.5)	.36
	General health	52.5 (20.3)	43.0 (16.4)	49.5 (18.0)	39.5 (21.0)	.02
	Change in health from 1 year ago	25.4 (32.4)	15.9 (27.1)	20.0 (32.3)	17.9 (22.3)	.50

^a^IP100: in-person exercise coaching 100%.

^b^IP50: in-person exercise coaching 50%.

^c^IP25: in-person exercise coaching 25%.

At baseline, we found that the control group scored higher on intrinsic motivational regulation than the 3 intervention groups (mean difference: 0.4-0.9; *P*≤.05). After the intervention, we found a between groups difference in 1 of the 8 health-related subscales for the SF-36. All intervention groups reported a greater improvement in self-perceived health in 1 of the 8 subscales of SF-36 (SF-36 domain: change in health from 1 year ago), than controls (IP100: 81.4, SD 17.5, *g*=0.82, *P*=.007; IP50: 80.9, SD 17.5, *g*=0.79, *P*=.01; IP25: 84.4, SD 12.4] *g*=1.14, *P*=.002; and controls: 66.7, SD 15.9; Table 2). No other intervention effects were found (Table 3).

**Table 2:** Differences between intervention arms in health-related quality of life at posttest.

Comparison				Mean difference		95% CI		Hedges *g*
**Health-related quality of life**								
	**Physical functioning**							
		IP100^a^ vs IP50^b^	5.28		–4.24 to 14.81		0.34	
		IP100 vs IP25^c^	2.50		–8.06 to 13.06		0.21	
		IP100 vs controls	3.46		–6.85 to 13.78		0.31	
		IP50 vs IP25	2.78		–13.35 to 7.77		–0.16	
		IP50 vs controls	1.82		–12.14 to 8.49		–0.11	
		IP25 vs controls	0.96		–10.32 to 12.24		0.07	
	**Role limitations due to physical health**							
		IP100 vs IP50	0.007		–19.93 to 19.92		0.00	
		IP100 vs IP25	6.44		–15.64 to 28.53		0.21	
		IP100 vs controls	2.58		–18.99 to 24.17		0.09	
		IP50 vs IP25	6.45		–15.64 to 28.54		0.20	
		IP50 vs controls	2.59		–18.99 to 24.17		0.08	
		IP25 vs controls	3.85		–27.45 to 19.73		–0.12	
	**Role limitations due to emotional problems**							
		IP100 vs IP50	1.90		–26.81 to 23.00		–0.05	
		IP100 vs IP25	5.91		–33.53 to 21.70		–0.15	
		IP100 vs controls	7.72		–34.71 to 19.25		–0.20	
		IP50 vs IP25	4.00		–31.62 to 23.61		–0.10	
		IP50 vs controls	5.82		–32.80 to 21.15		–0.15	
		IP25 vs controls	1.81		–27.68 to 31.31		–0.05	
	**Energy/fatigue**							
		IP100 vs IP50	5.71		–2.98 to 14.41		0.39	
		IP100 vs IP25	4.93		–4.71 to 14.58		0.36	
		IP100 vs controls	4.12		–5.30 to 13.55		0.37	
		IP50 vs IP25	0.77		–10.42 to 8.87		–0.05	
		IP50 vs controls	1.58		–11.01 to 7.83		–0.10	
		IP25 vs controls	0.81		–11.11 to 9.49		–0.07	
	**Emotional well-being**							
		IP100 vs IP50	3.54		–5.03 to 12.12		0.24	
		IP100 vs IP25	2.41		–11.93 to 7.09		–0.23	
		IP100 vs controls	1.08		–10.37 to 8.20		–0.09	
		IP50 vs IP25	5.96		–15.47 to 3.54		–0.40	
		IP50 vs controls	4.62		–13.92 to 4.66		–0.30	
		IP25 vs controls	1.33		–8.82 to 11.49		0.12	
	**Social functioning**							
		IP100 vs IP50	2.14		–18.26 to 22.54		0.07	
		IP100 vs IP25	0.34		–22.96 to 22.28		–0.01	
		IP100 vs controls	1.62		–20.47 to 23.72		0.05	
		IP50 vs IP25	2.48		–25.10 to 20.13		–0.08	
		IP50 vs controls	0.52		–22.62 to 21.57		–0.02	
		IP25 vs controls	1.96		–22.19 to 26.12		0.06	
	**Pain**							
		IP100 vs IP50	3.35		–15.18 to 21.89		0.11	
		IP100 vs IP25	9.11		–11.43 to 29.67		0.31	
		IP100 vs controls	1.53		–21.61 to 18.54		–0.05	
		IP50 vs IP25	5.75		–14.79 to 26.31		0.20	
		IP50 vs controls	4.89		–24.97 to 15.19		–0.17	
		IP25 vs controls	10.64		–32.60 to 11.30		–0.39	
	**General health**							
		IP100 vs IP50	9.09		–1.34 to 19.52		0.53	
		IP100 vs IP25	7.24		–4.32 to 18.81		0.52	
		IP100 vs controls	8.32		–2.97 to 19.62		0.58	
		IP50 vs IP25	1.84		–13.41 to 9.72		–0.10	
		IP50 vs controls	0.76		–12.06 to 10.53		–0.04	
		IP25 vs controls	1.07		–11.27 to 13.43		0.07	
	**Change in health from 1 year ago**							
		IP100 vs IP50	0.54		–10.10 to 11.19		0.03	
		IP100 vs IP25	2.94		–14.67 to 8.77		–0.19	
		IP100 vs controls	14.12		2.66 to 25.57		0.82	
		IP50 vs IP25	3.49		–15.28 to 8.30		–0.22	
		IP50 vs controls	13.57		2.04 to 25.10		0.79	
		IP25 vs controls	17.06		4.54 to 29.59		1.14	

^a^IP100: in-person exercise coaching 100%.

^b^IP50: in-person exercise coaching 50%.

^c^IP25: in-person exercise coaching 25%.

**Table 3:** Differences between intervention arms in exercise motivation, barriers to exercise, self-efficacy, and social support.

Comparison			Mean difference	95% CI	Hedges *g*
**Exercise motivation**					
	**Intrinsic regulation**				
		IP100^a^ vs IP50^b^	0.11	–0.45 to 0.68	0.14
		IP100 vs IP25^c^	0.15	–0.47 to 0.79	0.16
		IP100 vs controls	0.29	–0.32 to 0.91	0.34
		IP50 vs IP25	0.04	–0.58 to 0.67	0.04
		IP50 vs controls	0.17	–0.43 to 0.79	0.21
		IP25 vs controls	0.13	–0.53 to 0.81	0.14
	**Identified regulation**				
		IP100 vs IP50	0.10	–0.61 to 0.39	–0.15
		IP100 vs IP25	0.03	–0.52 to 0.59	0.05
		IP100 vs controls	0.24	–0.29 to 0.79	0.36
		IP50 vs IP25	0.14	–0.41 to 0.69	0.16
		IP50 vs controls	0.35	–0.19 to 0.90	0.48
		IP25 vs controls	0.21	–0.38 to 0.81	0.23
	**Introjected regulation**				
		IP100 vs IP50	0.17	–0.80 to 0.46	–0.18
		IP100 vs IP25	0.13	–0.57 to 0.83	0.12
		IP100 vs controls	0.01	–0.70 to 0.67	–0.02
		IP50 vs IP25	0.30	–0.40 to 1.00	0.29
		IP50 vs controls	0.15	–0.84 to 0.53	0.16
		IP25 vs controls	0.15	–0.60 to 0.90	–0.14
	**External regulation**				
		IP100 vs IP50	0.25	–0.72 to 0.20	–0.36
		IP100 vs IP25	0.008	–0.52 to 0.50	0.00
		IP100 vs controls	0.08	–0.59 to 0.41	–0.13
		IP50 vs IP25	0.24	–0.26 to 0.76	0.31
		IP50 vs controls	0.16	–0.33 to 0.67	0.21
		IP25 vs controls	0.08	–0.47 to 0.63	–0.11
	**Amotivation**				
		IP100 vs IP50	0.10	–0.35 to 0.15	–0.23
		IP100 vs IP25	0.03	–0.31 to 0.24	–0.12
		IP100 vs controls	0.03	–0.30 to 0.24	–0.11
		IP50 vs IP25	0.06	–0.21 to 0.34	0.15
		IP50 vs controls	0.06	–0.20 to 0.34	0.14
		IP25 vs controls	0.00	–0.29 to 0.29	0.00
**Barriers to exercise**					
	**Priority**				
		IP100 vs IP50	0.07	–0.25 to 0.39	0.13
		IP100 vs IP25	0.10	–0.25 to 0.46	0.21
		IP100 vs controls	0.06	–0.41 to 0.28	–0.16
		IP50 vs IP25	0.03	–0.32 to 0.39	0.06
		IP50 vs controls	0.13	–0.48 to 0.21	–0.27
		IP25 vs controls	0.17	–0.55 to 0.21	–0.37
	**Practical**				
		IP100 vs IP50	0.06	–0.17 to 0.30	0.17
		IP100 vs IP25	0.05	–0.21 to 0.32	0.15
		IP100 vs controls	0.13	–0.39 to 0.12	–0.38
		IP50 vs IP25	0.009	–0.27 to 0.25	–0.03
		IP50 vs controls	0.19	–0.46 to 0.06	–0.48
		IP25 vs controls	0.18	–0.47 to 0.09	–0.49
	**Health-related**				
		IP100 vs IP50	0.02	–0.23 to 0.28	0.08
		IP100 vs IP25	0.09	–0.38 to 0.19	–0.27
		IP100 vs controls	0.08	–0.36 to 0.19	–0.24
		IP50 vs IP25	0.12	–0.41 to 0.16	–0.31
		IP50 vs controls	0.11	–0.39 to 0.16	–0.29
		IP25 vs controls	0.008	–0.29 to 0.31	0.02
	**Affective-cognitive**				
		IP100 vs IP50	0.01	–0.20 to 0.16	–0.03
		IP100 vs IP25	0.05	–0.25 to 0.15	–0.24
		IP100 vs controls	0.09	–0.29 to 0.10	–0.36
		IP50 vs IP25	0.03	–0.23 to 0.16	–0.13
		IP50 vs controls	0.08	–0.28 to 0.11	–0.24
		IP25 vs controls	0.04	–0.26 to 0.17	–0.16
**Self-efficacy**					
	**Sticking to exercise**				
		IP100 vs IP50	0.10	–0.45 to 0.65	0.12
		IP100 vs IP25	0.04	–0.65 to 0.57	–0.05
		IP100 vs controls	0.17	–0.42 to 0.77	0.24
		IP50 vs IP25	0.14	–0.75 to 0.47	–0.14
		IP50 vs controls	0.07	–0.52 to 0.67	0.08
		IP25 vs controls	0.21	–0.44 to 0.86	0.24
	**Making time for exercise**				
		IP100 vs IP50	0.07	–0.68 to 0.54	–0.07
		IP100 vs IP25	0.24	–0.92 to 0.43	–0.26
		IP100 vs controls	0.20	–0.87 to 0.46	–0.23
		IP50 vs IP25	0.17	–0.85 to 0.51	–0.16
		IP50 vs controls	0.13	–0.80 to 0.53	–0.13
		IP25 vs controls	0.04	–0.69 to 0.77	0.04
**Social support**					
	IP100 vs IP50		1.45	–6.63 to 3.72	–0.17
	IP100 vs IP25		0.69	–6.43 to 5.04	–0.09
	IP100 vs controls		0.37	–5.97 to 5.23	–0.05
	IP50 vs IP25		0.76	–4.97 to 6.50	0.09
	IP50 vs controls		1.08	–4.52 to 6.69	0.13
	IP25 vs controls		0.32	–5.80 to 6.45	0.04

^a^IP100: in-person exercise coaching 100%.

^b^IP50: in-person exercise coaching 50%.

^c^IP25: in-person exercise coaching 25%.

## Discussion

### Principal Findings

This pragmatic RCT investigated the effectiveness of weekly in-person coaching compared with 2 different combinations of in-person and web-based coaching on motivation, barriers, self-efficacy, social support, and health-related quality of life among women with obesity. While no intervention effects were detected for most of the measured variables, a slight improvement was reported in self-perceived health in 1 of the 8 subscales of the SF-36. This improvement was consistent across all intervention groups when compared with the control group from before to after the intervention. However, it is important to note that the observed difference was limited to a single subscale of the SF-36, indicating that the interventions had only a minimal impact on overall health-related quality of life.

The improvement in the “change in health” subscale of the SF-36 across all intervention groups suggests that combining in-person and web-based exercise coaching may contribute to enhanced self-perceived health among women with obesity. This is consistent with a previous RCT involving 143 inactive women with excess body weight, which found that those who received in-person exercise coaching by a personal trainer reported improved self-perceived health after 12 weeks of intervention [42]. Interestingly, this RCT also found that those who exercised alone reported better self-perceived health compared with the control group [42]. This may be due to the fact that regular physical activity, even at low levels (≥1 day/week), has been shown to improve self-perceived health [57]. In a previous longitudinal prospective study among novice exercisers in a fitness club setting, regular exercise in the fitness club (≥2 days/week) was associated with high self-perceived health (odds ratio 3.53, 95% CI 1.60-7.82; *P*=.002) [58]. Therefore, regular exercise itself may improve self-perceived health, emphasizing exercise as a key factor in overall well-being. Although the use of a personal trainer may not directly influence self-perceived health, it seems to bolster exercise adherence, thus supporting long-term health benefits. This appears to be consistent regardless of in-person or web-based coaching, as we did not find differences in exercise frequency between the 3 intervention groups. However, since this improvement in our study was limited to just 1 of the 8 subscales of the SF-36, further research is necessary to determine whether this represents a true and clinically significant effect.

The intervention did not demonstrate significant effects on exercise motivation, which may be explained by several factors. One key factor is the baseline differences in intrinsic motivational regulation between the control group and the intervention arms, with the control group having higher intrinsic motivation at baseline. This disparity may have created a ceiling effect, limiting further improvements in motivation both within and between groups. However, all groups scored between 1.7 and 2.6 on the 5-point scale, indicating room for improvement, irrespective of the initial differences. In addition, variations in the coaching approaches of the personal trainers, which were not controlled for in this study, could influence exercise motivation. According to self-determination theory (SDT), interactions between the personal trainer and the client may enhance motivation for exercise by satisfying the basic psychological needs (autonomy, competence, and relatedness) [59]. Personal trainers who adopt a need-supportive coaching approach, such as providing meaningful choices, acknowledging client feelings, and offering constructive feedback, may foster these needs and thereby improve exercise motivation [30]. For instance, one study investigating the effects of an SDT-based physical activity program among adults with obesity (BMI ≥40 kg/m^2^) found that perceived autonomy support and the satisfaction of basic psychological were positively associated with increases in autonomous motivation and negatively associated with controlled motivation and amotivation [60]. On the other hand, certain coaching approaches, which include elements like guilt-inducing language or not fully considering the clients’ preferences, may inadvertently impact these psychological needs and reduce motivation [30].

By offering scheduled appointments, free follow-up sessions, and access to exercise equipment, the intervention effectively minimized many logistical and financial barriers to exercise. However, we found no changes in exercise barriers, with all intervention groups reporting high scores on barriers such as “priority” (lack of time and energy) after the intervention. These findings are consistent with previous studies [52,61-63], suggesting reducing logistical obstacles alone may not be sufficient to overcome exercise barriers. This highlights the importance of addressing more individual factors, such as personal priorities and attitudes toward exercise. Overcoming these barriers likely requires coaching strategies that go beyond logistical support to include more personalized approaches tailored to each individual’s needs and circumstances [64,65].

The lack of effect on self-efficacy in our study is in line with 2 previous RCTs, which reported no differences between in-person and phone-based exercise coaching [66,67]. While one cross-sectional study found an association between in-person coaching and improvement in self-efficacy compared with web-based coaching [68], this was not reflected in our results. One potential explanation for the varying results could be the high baseline self-efficacy scores across all groups, indicating that participants who already had high self-efficacy may have had limited room for further improvement [31,37]. Nevertheless, it is important to note that all intervention arms in our study exercised significantly more than the control group after the intervention, even without a corresponding increase in self-efficacy. This suggests that improvements in exercise adherence may occur independently of self-efficacy changes. Future research should explore additional support mechanisms, such as coaching approaches grounded in behavioral theories, to further enhance exercise participation. For instance, one study that applied social cognitive theory–based coaching, which focuses on psychological factors such as self-efficacy, found that women in the coaching group had higher exercise adherence (49% versus 31%) compared with the control group, alongside improvements in self-efficacy [69].

The measurement used in our study included perceived social support from friends and family, unfortunately not accounting for potential support the participants might have experienced from their personal trainer. Including data on social support from personal trainers would have offered a more comprehensive understanding of participants’ support networks, while also highlighting the trainers’ role in promoting exercise adherence. In addition, it could have provided valuable insights into the dynamics of the trainer-participant relationship. However, low social support scores, both before and after the intervention, indicate a lack of sufficient encouragement from the significant others. This finding is important, as social support from family and friends is a key factor that consistently influences exercise adherence [25,26].

Our findings align with the broader literature, highlighting the complex relationship between exercise interventions and psychosocial outcomes among individuals with obesity [70,71]. For instance, Pilates, yoga, tai chi, and qigong have demonstrated potential for improving mental well-being in populations living with obesity [70,71]. However, the evidence regarding their direct effects on broader psychosocial factors remains limited. Further, a recent review showed clear evidence of combined aerobic and resistance training in improving key health indicators, including glycemic control and mental health, among individuals with obesity and type 2 diabetes [72], while our study did not demonstrate significant changes in health-related quality of life across all dimensions. The small improvement in the “change in health” subscale may reflect the cumulative benefit of structured exercise on self-perceived well-being. These results also support that regular exercise may enhance specific quality-of-life dimensions, even without observable changes in other psychosocial variables.

Finally, while this study investigated the effect of hybrid coaching models, targeted exercise regimens, such as high-intensity interval training, are shown to have the potential to improve psychosocial factors, including enhanced self-perceived health [73]. Further, exercise modalities like Pilates and yoga could also complement exercise coaching to improve psychosocial factors, since these approaches offer a less demanding, more inclusive alternative, particularly for individuals facing physical or psychological limitations [70,71,74]. Future research may integrate such modalities within hybrid coaching models to evaluate their combined impact on psychosocial outcomes.

Increasing exercise participation among the population living with obesity could significantly impact public health. Since motivation, barriers, self-efficacy, and social support are key to exercise adherence [25,75], research should explore how various coaching approaches affect these factors. For instance, SDT-based coaching may help personal trainers create a more supportive and motivating environment, improving adherence [30]. Despite the lack of effect on motivation, the intervention’s potential for positive impact lies in highlighting the importance of SDT-aligned coaching strategies, which could be key to improving motivation in future interventions targeting populations living with obesity. By integrating behavioral theories into coaching interventions, researchers may better identify strategies to improve motivation, reduce barriers, and strengthen self-efficacy, ultimately promoting sustainable exercise habits and improved health outcomes [76]. While the intervention did not reduce self-reported barriers, it minimized external challenges, highlighting the value of interventions that address personal barriers. Last, although in-person and web-based coaching showed similar effects, the potential cost-effectiveness and accessibility of web-based coaching make hybrid or fully web-based approaches appealing alternatives for supporting exercise adherence among populations living with obesity. This is especially relevant given that logistical and financial barriers often limit consistent participation in in-person coaching [43,44,46]. While our RCT did not demonstrate differences in psychosocial outcomes, it shed light on the feasibility and value of hybrid coaching models to support exercise adherence in obese populations.

Our study is one of the first global pragmatic RCTs of its kind, with a long intervention period and a unique catchment area, including a nationwide intervention spanning both urban and rural regions of Norway. The inclusion of 23 experienced personal trainers, each with at least 3 years of experience, further enhances the robustness and generalizability of our findings in a real-world setting. Another strength is the hybrid approach, combining web-based coaching with in-person sessions. This approach reflects the contemporary trends in exercise supervision and meets the evolving needs of participants in modern society. Our study also contributes to the United Nations’ Sustainable Development Goal 5 by focusing on obese women and addressing psychosocial factors through in-person and web-based exercise coaching, thereby supporting efforts to improve health equity and empower women. Finally, we used a range of validated questionnaires to measure the psychosocial outcomes, including motivation (BREQ-2), self-efficacy (Self-Efficacy Survey), social support, and health-related quality of life (SF-36), providing a comprehensive assessment of the psychosocial effects of the intervention.

### Limitations

Some study limitations should be considered when interpreting our results. We unfortunately had a notable dropout rate, with only 64% (120/188) of participants completing both the baseline and postintervention questionnaires. However, no significant differences were found between those who completed the study and those who dropped out in terms of baseline data. Further, the 20-week intervention period may be too short to fully assess the intervention’s effects, particularly on psychosocial factors such as self-efficacy, motivation, and health-related quality of life, which often require longer durations to reveal changes or differences. Finally, while this study was conducted in Norway, limiting direct global generalizability, culture and societal similarities between Norway and other Western countries suggests that our findings could apply to similar populations and contexts. Further, the lack of a priori power calculation is another limitation of this study. The sample size was determined based on practical considerations rather than statistical power affecting the robustness and generalizability of our findings, as the study may be underpowered to identify small effects. Last, the Shapiro-Wilk test was used to evaluate whether our data met the assumption of normality. While this test is widely recognized for assessing normality, it is sensitive to sample size and outliers, which may limit its ability to fully characterize data distributions. In our study, as many as 120 participants completed both baseline and postintervention questionnaires. Thus, we believe the results of the Shapiro-Wilk test align with the assumption of normality.

### Conclusions

This study indicates that both in-person coaching and 2 combinations of in-person and web-based coaching may lead to a slight improvement in self-perceived health, by higher scores in 1 of the 8 SF-36 subscales. However, these observed improvements are preliminary and may not generalize to broader health or psychosocial domains without further evidence. No significant effects were found on motivation, barriers, self-efficacy, social support, or other health domains. Further research is needed to fully understand how we can increase the ability to maintain regular exercise among women with obesity.

## Appendix

Multimedia Appendix 1 CONSORT-eHEALTH checklist (V 1.6.1).

## Abbreviations

BREQ-2: Behavioral Regulation in Exercise Questionnaire-2
IP25: in-person exercise coaching 25%
IP50: in-person exercise coaching 50%
IP100: in-person exercise coaching 100%
RCT: randomized controlled trial
SDT: self-determination theory
SF-36: 36-Item Short Form Health Survey

## Data Availability

The datasets generated or analyzed during this study are available from the corresponding author on reasonable request.
